# “My body…tends to betray me sometimes”: a Qualitative Analysis of Affective and Perceptual Body Image in Individuals Living with Endometriosis

**DOI:** 10.1007/s12529-022-10118-1

**Published:** 2022-09-08

**Authors:** Katherine Sayer-Jones, Kerry A. Sherman

**Affiliations:** 1 Centre for Emotional Health, School of Psychological Sciences, Macquarie University, Sydney, Australia; 2 Centre for Emotional Health, School of Psychological Sciences, Macquarie University, Sydney, NSW 2109 Australia

**Keywords:** Body image, Endometriosis, Qualitative, Chronic illness, Gynecology, Thematic analysis

## Abstract

**Background:**

Endometriosis is a chronic reproductive disease manifesting in physical symptoms including pain, abdominal swelling, altered bowel and bladder function, and fatigue. These symptoms potentially threaten body image regarding subjective perceptions of functional, appearance, and sensory aspects of one’s body. The aim of this study was to qualitatively understand how endometriosis impacts on affective and perceptual aspects of body image.

**Method:**

Participants (*N* = 40) were recruited through endometriosis consumer organizations. In an online survey, participants completed demographic and health history questions, then provided written narratives about body image–related impacts of their endometriosis in response to open-ended questions. These data were thematically analyzed using the template approach.

**Findings:**

The majority of participants (M_age_ = 28.3 years) were employed part-time, diagnosed on average for 4.2 years, and reported pelvic pain and bloating, fatigue, and nausea symptoms. Thematic analysis yielded three themes including My Body is a Barrier, Needing to Hide Myself, and Body as Healer and Teacher, all of which reflected affective and perceptual aspects of body image.

**Conclusion:**

These findings highlight wide-ranging body image–related impacts of endometriosis, suggesting the need for targeted interventions to address these concerns.

## Introduction

As a chronic disorder, endometriosis affects 10–15% female-identified individuals of reproductive age globally [1, 2]. It is regarded as a systemic inflammatory condition that is predominantly characterized by pelvic pain, including dysmenorrhea (menstrual pain), dyspareunia (painful sexual contact), and dyschezia (defecation pain) [3]. Abdominal bloating, fatigue, gastrointestinal distress, and female infertility [4] are also commonly experienced. Quality of life and mental health are diminished in those living with endometriosis [5, 6]. Moreover, this psychological distress is associated with diagnostic delays, symptom severity [7], and low self-image [6, 8].

Despite the physical and psychological impacts of endometriosis [9], research investigating body image impacts utilizing the broader body image conceptualization in a chronic disease context is lacking. The construct of body image comprises four distinct domains relating to cognitive, affective, perceptual, and behavioral domains [10]. Negative body image is regarded as distressing when it involves intrusive thoughts, negative changes to self-concept, and social aversion [11]. In the face of a serious chronic illness, such as endometriosis, that is characterized by high symptom burden and psychological distress, the affective and perceptual domains of body are highly relevant. That is, in an emotionally charged situation of living with and managing the somewhat unpredictable and changing symptoms of endometriosis, the way that an individual responds emotionally about their body (i.e., affective body image) [12] is likely to be a key factor underlying psychological adjustment. Another aspect of perceptual body image is appreciation of the body’s functionality [10, 11, 13–15], a positive aspect of body image that reflects how an individual respects and appreciates their body as it is, rather than what it could or should be [16]. Functional appreciation is likely to be an important aspect of body image in endometriosis, given the need to live with extensive changes and bodily impacts on menstrual and sexual functioning, fertility, and pain associated with this condition [17]. Notably, adverse body image impacts do not necessarily have a one-to-one correspondence with disease stage or progression, with extensive body image impacts sometimes evident in early stage disease [18].

Emerging research has identified considerable negative impacts of endometriosis on body image [19, 20], particularly for those with extensive disease [21] and younger adults [19]. Qualitative research [22] highlights widespread negative body image impacts linked to hormonal treatment weight gain and scarring from surgery, with concealment practices (e.g., wearing loose clothing) utilized as a means of coping with these bodily changes. Body dissatisfaction, a proxy for negative body image, has been associated with infertility and dyspareunia in endometriosis [22], consistent with findings of research investigating body image distress in gynecological cancer [23]. Moreover, more negative body image has been associated with increased self-criticism in endometriosis populations, a factor that is incompatible with body functionality appreciation [20]. Another factor likely to exacerbate negative responses to an endometriosis-affected body is the nature of endometriosis being encased within menstrual social taboos that may increase vulnerability to body image distress due to diminished social supports [24]. Taken together, this emerging literature indicates widespread body image–related distress in individuals living with endometriosis. Yet, a detailed understanding of body image in this context is lacking as prior research has tended to treat body image holistically as a single construct (e.g., body appreciation [20] or body dissatisfaction [21]), without consideration of the specific domains. Given the likely importance of affective and perceptual (sensory and functional appreciation) aspects of body image to individuals living with endometriosis, the aim of this study was to gain an in-depth, qualitative understanding of how these domains are impacted by this condition. We addressed the research question of what are the affective and perceptual body image impacts of living with endometriosis?

## Method

### Participants

Between April and August 2020, female individuals and one non-binary individual with (self-reported) endometriosis diagnosis were invited to participate in the study through three sources: online postings via Australian endometriosis consumer organizations (Endometriosis Australia, EndoActive, Endogram), first-year psychology student research pool, and a flyer distributed through an endometriosis gynecology clinic. Student participants received course credit; no other incentives were provided. This multi-modal recruitment strategy aimed to increase the potential for sample diversity. Eligibility criteria included being a resident in Australia, at least 18 years old, competent to read and write about their endometriosis experiences in English, and internet connected.

### Procedure

After accessing the study link provided in the invitation, participants provided online consent and completed a survey on the Qualtrics platform. Information pertaining to demographic (age, gender, education, ethnicity, employment status, relationship status, parity) and medical (diagnosis method, time since diagnosis, endometriosis symptoms, endometriosis treatment) characteristics was initially collected. Then, participants responded to six open-ended questions designed to elicit written qualitative responses regarding their experiences, concerns, and issues related to endometriosis and their subjective body-related perceptions (i.e., body image) based on prior body-image research (see Table 1) [25]. An additional open-ended question at the end of the survey allowed participants to share any further information regarding their experiences. The sample size (*N* = 40) was deemed sufficient for a qualitative analysis [26]. Ethics approval was granted from the Macquarie University Human Research Ethics Committee (HREC number: 52020565115411).

**Table 1:** Short-answer survey questions and follow-up prompts

1) Can you give me a brief summary of the history of your Endometriosis?
- How does Endometriosis affect your everyday life?
- What treatment have you had? In your view, how successful has your treatment been?
- What would the best possible outcome of treatment be?
- What do you do on a daily basis to manage your Endometriosis?
- What do you do on a daily basis to manage your Endometriosis?
- Would you say you are in control of your Endometriosis?
2) How does Endometriosis affect your relationship with your body?
- Did Endometriosis cause barriers for you to be your real self?
- Has there been a time when this was more/less the case?
- Have there been any positive effects?
- What was the worst period? Have things got better over time?
- How does this differ, do you think, to how other women experience their bodies?
- How does this affect your well-being?
- Does this vary depending on symptoms?
3) How has Endometriosis altered your sense of attractiveness?
- How does it affect how you feel about your body?
- Do symptoms of Endometriosis make you feel more or less attractive? In what way?
- Has it affected your experience of how potential partners/your current partner perceived you?
4) Could you tell us if and how Endometriosis has affected how you dress and present yourself?
- Do you adjust how you dress due to symptoms?
- Does this fluctuate over time? If so, what determines this?
- Does this affect how you feel about your body?
- How do you experience the world in these instances?
- Does this vary at different times? What determines this?
5) How has Endometriosis impacted how you view your own body relative to others?
6) Women with other gynecological conditions have cited body acceptance as an experience they learnt through their experience of having their condition. Could you tell us about this experience if this is something you relate to?
- Has your Endometriosis changed your appreciation of your body?
- Have there been benefits to having an Endometriosis diagnosis in how you understand your body’s function?
7) This is the final item and we would like to know if there is anything you would like to say about these topics that we have not already asked you about?

### Data Analysis

These qualitative data were analyzed by two researchers: (1) a female postgraduate student provisional psychologist (author 1); and (2) a female senior research academic with extensive experience in health-related qualitative and quantitative research and who also has lived experience of endometriosis (author 2). Data were thematically analyzed using a template approach [27] guided by the six-stage [28] inductive method entailing (1) familiarization with the whole dataset, where the authors each read these data once, then repeatedly read the whole data set while actively searching for patterns and meaning by keeping notes; (2) the authors separately generated initial codes through the process of organizing these data into meaningful clusters using qualitative software NVivo (Version 12.4.0); (3) the authors compared codes and identification of similarity between codes to form initial themes; (4) author 1 compared the overlap between codes and consolidated them, and then shared this with author 2 who provided feedback; (5) together both researchers iteratively named each theme, defined the singular focus of the theme, how each theme was distinct or built upon previous themes, and how it answered the research question; and, (6) the final integration of results resulted in the process of writing the analysis and selecting salient quotes, which was completed primarily by author 1 and reviewed by author 2. Notably, the research question guided each stage of analysis. As previously applied to qualitatively understanding reproductive health topics [29], this analysis adopted a critical realist approach [30]. This approach enabled the researchers to accept that the participants’ experiences were reality, acknowledging how individuals make sense of their endometriosis experiences and body image while examining the broader social context in which these experiences arose. We utilized bracketing to capture the participant experience as illustrative quotes of the identified themes.

## Findings

Sample characteristics are provided in Table 2. Three themes were developed from the qualitative data analysis associated with the affective and perceptual aspects of body image: My Body is a Barrier, Needing to Hide Myself, and Body as Healer and Teacher. Similarities and differences across participant’s accounts were explored. De-identified illustrative quotes are presented, linked to a participant via an ID number, prefixed with a P for participant.

**Table 2:** Demographics and medical characteristics of participants

Characteristic	Frequency (*n*)
Age, years	
Mean (range)	28.3 (18 to 50)
SD	9.39
Gender	
Female	39
Non-binary	1
Relationship status	
Single	42.5% (17)
Partnered, living separately	27.5% (11)
Partnered, living together	30% (12)
Highest level of education obtained	
High school	30% (12)
Certificate or diploma	22.5% (9)
Undergraduate	27.5% (11)
Postgraduate	20% (8)
Employment	
Unable to work	5% (2)
Unemployed	15% (6)
Less than 38 h per week	55% (22)
38 h or more hours per week	25% (10)
Method of diagnosis*	
Surgery	52.5% (21)
Clinical	25% (10)
Diagnostic imaging	30% (12)
Self	5% (2)
Treatment method (current or previous)*	
Surgery	52.5% (21)
Hormonal (pill, IUD)	87.5% (35)
Pain medication	40% (16)
Allied health**	25% (10)
Time since diagnosis (years)	4.23
Number of symptoms	
Symptoms*	
Pelvic pain	95% (38)
Bloating fullness	87.5% (35)
Fatigue	77.5% (31)
Nausea	60% (24)
Painful sexual contact	47.5% (19)
Abnormal bleeding	50% (20)
Gastrointestinal	25% (15)
Headaches	32.5% (13)

*SD* Standard deviation

^*^Participants counted for all strategies selected

^**^Allied health included psychology, counselling, acupuncture, homeopathy, dietetics, nutrition, Chinese medicine, physiotherapy, and personal training

### Themes

#### My Body is a Barrier

Endometriosis symptoms were perceived as burdensome for the majority of participants, with the unpredictability and enduring nature of the disease causing lowered bodily appreciation. As a chronic disease with intermittent symptoms and progressive trajectory, endometriosis severely impacted participants’ daily lives, temporarily immobilizing them, reducing attendance at workplaces and social events, and leading them to feel distressed: “Endometriosis has gotten in the way of many things in my life, causing me to cancel plans, forcing me to take days off work etc. I do not have a good relationship with my body as it does feel like it tends to betray me sometimes” (P30). This emotive betrayal was expressed across many accounts, with endometriosis symptoms being seen to disappoint participants’ expectations of how their body needed to function. As P31 described, “I don’t trust it. I have lost my ability to view it as a dependable machine that serves me positively.” Feeling let down by the need to reduce capacity and commitments was integral to the perceived betrayal that required enormous effort to maintain normal routine when symptomatic, as P36 indicated, “Sometimes I have to dig deep and just keep going, however it’s incredibly hard.”

Pain dominated descriptions of individuals resenting their body’s need for reduced activity and was associated with labelling the self as disabled and feelings of grief associated with lost opportunities: “My body has now become a prison. I used to be extremely active ……. Now activity causes pain and has rendered me a couch potato. I miss being active” (P3). Frustration was evident in having to make daily choices based on anticipation of trigger activities (e.g., exercise or foods) that could lead to “flare ups”; P23 described “I have to think about when and if I should go to the gym or do yoga based on what I need to do work and life wise. As often these activities increase my pain and bloating.” Loss of body functionality was evident in several participants’ accounts of infertility, with fertility challenges or hysterectomy intervention impacting their gender identity and ability to fulfil life choices, “Makes me feel like I can’t live up to the expectation of society in terms of bearing children” (P22).

Endometriosis was regarded as an “other” or malign force that hindered how participants perceived their bodies were meant to work, reducing control and body functionality appreciation. P12 indicated, “I have the feeling that my whole body is affected by endometriosis and kind of stopped working in the appropriate way” and P18: “I find my body extremely frustrating mostly because it continues to grow endometrium-like cells outside my uterus and no one can tell me why or what to do to stop it.” Moreover, the enduring, intermittent symptoms disrupt participants’ ability to mentally and physically synchronize with their bodies, as P20 noted, “The return of symptoms is by far the hardest part of the process for me on my body.” Many grieved the ability to make flexible choices, which created a sense of hopelessness. Some participants reported dissociating their physical function from mental desires as a coping mechanism, as P2 said, “I disassociated from my body and myself. I survived.”

With half of the sample under the age of 30 years, many reported that the need for sporadic rest and reduced activity forced upon them by the severity of endometriosis symptoms experienced was distressingly incongruent with their peers’ physical vitality and reflected low functionality appreciation: “Endo definitely makes me feel somewhat more ‘broken’ than other people my age. For instance, if I was to say, ‘my back is sore today’ (due to endo), others have remarked that I’m too young to have a bad back. It’s hard not to feel like I have a ‘dud’ body when others my age can do so much more” (P11). Endometriosis symptoms were distressing when contextualized as a hindrance to being seen, heard, and understood by peers which resulted in shame-inducing social isolation, “I felt alone and embarrassed as none of my friends could relate to what I was feeling” (P19). For older participants, they reflected on the enduring consequences of endometriosis as an emotional process of grieving, especially when the disease trajectory meant loss of time and opportunity spent on desired plans: “I think physical is only one aspect. Endometriosis is a mind game. The strength required to navigate the path, the disappointments, the surgeries, the infertility, the cost, the loss. The pain is a constant reminder of how much your body has been overtaken by these weeds regardless of all the myths of treatment” (P31, age 50).

#### Needing to Hide Myself

Endometriosis created a multitude of disruptive experiences and negative emotions for participants including feeling unattractive, and feeling ongoing pressure to physically conceal themselves, due to shame and embarrassment associated with their symptomatic bodies. Participants reported that their modifications exceeded adjustment for physical sensations alone (e.g., modifying clothing choices due to alleviating pressure on the pelvis) and reflected an urge to camouflage their appearance due to believing they looked culturally transgressive, that is, having a non-normative body. These concerns occurred in public and private contexts, with participants expressing that their perception of their physical appearance negatively shifted in the eyes of others when experiencing a “flare up.”

Participants described changing clothing choices when symptoms were present, with the aim to conceal changed body parts and minimize discomfort. In particular, abdominal bloating during “flare ups” or menstruation was emotionally laden reflecting affective aspects of body image perceptions, described as embarrassing and socially undesirable, “endo belly” or “looking pregnant”: “Some pants…have become a problem because my belly got very sensitive and I get more pain if there is pressure of clothes… If I get a flare up I look like I’m pregnant, so I often prefer to wear loose clothes” (P12).

For some, these informal or conservative clothing choices during endometriosis symptoms diminished their self-image and pleasure in their self-presentation, “I love color and wearing nice clothes to cheer myself up, but especially on bad days this isn’t possible” (P6). The need to dress comfortably was akin to dressing casually, which was viewed as compounding negative psychological effects and was linked with lowered self-confidence, P36 reported: “Days where there is a lot of discomfort and pain I tend to wear loose, baggy clothing such as trackies, which in turn makes me feel slouchy and lazy, and I don’t feel very confident at all.”

Additionally, participants expressed affective aspects of body image regarding frustration and fear of humiliation in public, which resulted in actions to accommodate their unpredictable symptoms, such as pre-planning clothing choices. P1 spoke for many: “I think about what I should wear based on how potentially bloated I might become over the course of the day. I find getting dressed up in the evenings the hardest… (because so many foods trigger me) becomes a very loaded and anxious experience.”

The desire to live in a healthy body that ascribed to feminine norms and did not require dress code adjustment was evident in individuals’ accounts envying unaffected women, as P5, “my Endo Belly really bothers me, and I want to be the same size and shape all over, like other women.” Moreover, participants like P29 expressed that their internal comparisons to others were problematic as it resulted in viewing themselves as lesser and led to negative feelings of insecurity, “I suffer from a lot of comparing and wishing I was just normal. I always feel insecure about my body.”

In contrast, several participants exemplified a more positive approach, stating that they were actively not comparing themselves to others: “There is no point in comparing my body to others. My symptoms are never permanent” (P15). Further variability existed in these accounts, P11 described gratitude that her life circumstances permitted her to not ascribe to a dress code that would hinder their capacity to dress according to physical fluctuations: “Thankfully I am in a workplace where I don’t need to be too formal or have a uniform.” Within these accounts, the desire to withdraw socially from others for the purposes of healing and managing pain was an instance of appearance related assimilation to culturally fit in. For some, being surrounded by supportive relationships mitigated their endometriosis-related appearance concerns, “It depends who I’m with though—with friends/partners I feel okay, but at parties I might be a bit more conservative” (P20). In contrast, some participants indicated that their appearance was not a concern at all.

Self-consciousness and appearance concerns related to the self-perception of being unattractive often extended to participants experiencing adverse emotional reactions of feeling distressed. In public, these concerns meant participants felt physically and sexually unattractive to others, and consequently, wanted to remain hidden: “I don’t really want anyone to see the shape of my body because I know it’s not a true reflection of my body and look” (P24). Privately, participants described their endometriosis as affecting their sexual intimacy which was experienced as an acute challenge when physical appearance altered during flare ups. The sensory experience of pain had a negative affective impact on individuals’ perceptions of themselves in the way they anticipated others would not intimately desire them, “It’s hard to find confidence or see how anyone could find you attractive when you are a walking ball of pain and yukky symptoms” (P8). For some, feeling unattractive negatively impacted their ability to feel comfortable and seek intimate connection, “(I feel) less attractive. I now find it very hard to date or being intimate with others as I’m not happy with the scars and the way I look” (P14). Although symptoms could be concealed to the public eye, it was the prospect of nudity revealing scarring, bloating, and bleeding, where self-doubt about attractiveness centered. P20 reported endometriosis had negatively impacted the perceptions of her body and subsequently her comfort in engaging in intimacy: “It’s impacted how attractive and comfortable I feel in my body. It’s had a flow on effect to how I feel in my intimate relationship. The bloating … and difficulty in losing weight is a challenge for me.”

For those with intimate partners, several were careful to separate their self-perceptions of their attractiveness and what their partners reported. As P24 said, “I feel unattractive, as I’m bloating, and I feel fat… My current partner doesn’t seem to notice it’s more of my own reflection of myself.” Private self-confidence and perception of attractiveness was evidently privileged over partner’s reassurances. Embarrassment was frequently experienced before and during disclosure to intimate partners about perceived difficulties. While partner support appeared to reduce participants’ negative perception of their bodies as shameful or problematic, it did not negate endometriosis being positioned as a disruptive and distressing experience both individually and towards interpersonal relationships. As P1 expressed: “Endo impacts everything. I described it to my husband as if we have a third person in our house. She’s a stroppy unpredictable teenager. You can try ignoring her but she’s there and she’s gonna go [‘mess up’ expletives] up when you least expect it. It’s a burden to have to live like this… for both of us. Endo doesn’t just impact the one who has it.”

#### Body as Healer and Teacher

Learning to live with endometriosis was themed by accepting the variable reality of the disease symptoms and appreciating what the body can do, that is functionality appreciation, as distinguished from denying or rejecting the way the disease impacted the body.

Diagnosis served as a powerful, vindicating event for participants who reported that the medical clarification affirmed their long-held belief that their menstrual problems were atypical. This information helped restore trust in their intuition and in their understanding of their own body and its functioning, leading to greater functionality appreciation. As P22 indicated, “When my surgeon listed off a raft of symptoms I’d probably experienced because of where the endo was located I felt such relief and was able to accept my body and pain more.” Participants recounted that post-diagnosis education describing the various ways in which endometriosis manifests assisted them by changing the perception of their bodies. Specifically, having greater awareness of typical symptoms of endometriosis enabled them to perceive fewer negative sensory and affective (i.e., symptom-related anxiety) bodily impacts. As P20 described, “I used to worry a bit about what caused the really sharp intense pains I experienced and if there was some kind of damage being done internally. Since finding out about having endo I’ve felt less concerned about it.”

Furthermore, diagnosis provided individuals with the language to talk about their experience which relieved their emotional isolation and altered their perception of their endometriosis-affected body, expressed by P2, “My diagnosis changed everything. My complaints/symptoms were finally taken seriously.” This reprieve from denial meant individuals’ symptoms were shifted to being contextualized within the concept of endometriosis as a disease; this knowledge served as healing, empowering participants to understand their bodies, as opposed to interpreting their symptoms as psychosomatic dramatization.

Markedly, some participants expressed that the temporary nature of endometriosis symptoms made them tolerable and increased their ability to cope, increasing their sense of body functionality appreciation and overall acceptance. Accounts of acceptance varied along a trajectory. P13 described moving away from hatred: “I went through a pretty bad ‘hating’ stage where I just felt like my body had let me down and it ‘wasn’t fair.’ I’m almost on the other side of this now, but definitely not at the ‘body acceptance’ stage yet.” In other accounts, an attitude of radical acceptance aided coping but did not equate to approval; negative symptoms could co-exist as part of the body in a way that did not discount positive and sustaining aspects. These participants described intentionally removing blame from their body for causing their disease. Within these accounts a shift to body acceptance language and functionality appreciation characterized descriptions. As P5 expressed: “Do my best to love my body, as I have realized over the years it’s not my body’s fault this is happening. I do feel less of myself though, especially when pain, fatigue, nausea levels are high. Endo has helped me appreciate my good days more.” Some participants viewed the cyclical characteristic of the disease as beneficial, as symptoms were intermittent and variable in severity throughout their cycle. Others expressed gratitude and functionality appreciation that their bodies coped and adapted, as imbued in P4’s account that positioned pain as a messenger of something organic that demanded prioritization: “I feel my body is amazing in the ability it has to keep going! I appreciate it for letting me know it needed help and surgery. All these signs and pain are because I have a condition that needs to be addressed. I appreciate the good days way more and I am less focused on appearance.”

Most of those surveyed utilized a combination of methods to alleviate symptoms, describing a process of trial and error, where the path to improved symptoms often involved increased symptom severity before finding an intervention that worked, and departures from mainstream treatments. A sense of control characterized daily lifestyle choices, greater appreciation for one’s body, and pride in acknowledging these efforts as a means of improving health. Several participants described cancelling or delaying surgery after seeing improvement via lifestyle strategies, which also fostered psychological autonomy, as P28 reported: “I was scheduled to have surgery but decided against it so I’m now just on the pill and saw a naturopath for a year… I’m in control now as I know what I need to do to help through years of learning.” These participants managed their symptoms with pragmatic flexibility reflecting reality acceptance skills. As P25 described, “I’ve learnt a lot about my body through trial and error which is good for me in the long run.” Gratitude for the knowledge gained from ongoing management reflected individuals’ improved relationship with their bodies.

In several accounts, surgery was positioned as a short-term fix, with symptoms returning within a year of the invasive procedure. Disappointment when both surgical and non-surgical strategies were unhelpful produced distress and disheartenment. It was psychologically distressing when symptoms re-emerged despite participants’ best management efforts, described by P18: “I go through peaks and troughs with the management. Doing lots of good things for my health (diet, exercise, acupuncture) makes me feel great but when my symptoms return every 2–4 weeks I feel so defeated.” Participants’ intermittent disappointment did not negate the importance of management but embodied the negative emotions of cyclical sorrow when symptoms returned when longing for respite. Accounts frequently described the effort required to manage their endometriosis as an unrelenting but crucial process in their lives. Several participants reported adverse psychological effects of hormonal-based interventions. This group actively prioritized stable mood and “mental health” over intermittent endometriosis symptoms.

Despite the majority of participants describing adverse body image impacts of endometriosis, some indicated they were not aware of any such difficulties arising from their condition. For example, P17 indicated that “I don’t feel in any way negative about myself and my attractiveness because of my endometriosis” and P32 reported “I’ve never really associated my attractiveness with endometriosis, or felt that it challenges me in that way. The only thing that makes me somewhat self conscious is the bloating and maybe facial breakouts, but both of those things can be linked to the hormonal changes that accompany regular period cycles.” Here, P32 is rationalizing these appearance changes as being a normal part of menstruating, not an insurmountable endometriosis-related challenge.

## Discussion

This qualitative analysis provides confirmation that endometriosis challenges the ability to have a positive relationship with one’s body, in terms of affective and perceptual aspects of body image. In particular, when symptomatic, endometriosis creates a sense of malaise with negative impacts on perceptions of body appearance, sensations, and appreciation of body functionality, as well as negative affective responses to one’s body.

The *My body as a barrier* theme characterized endometriosis as a constant force that served to diminish participants’ functional appreciation of their bodies. Participants viewed the symptomatic disease as a burden limiting their desired daily functioning in domains including work attendance, social life, exercise, and study, corroborating findings that endometriosis-related role disruption results in frustration, disappointment, and demotivation [31]. This is consistent with prior endometriosis [5, 6, 22, 32, 33] and chronic illness [34] research, reflecting the concept of experiencing disruption versus restoring continuity [8]. Our findings extend this theoretical concept by highlighting how endometriosis creates the affective response of a sense of grieving for a desired, but seemingly unattainable, body image trajectory. That is, a dissonance exists between expectations and reality of their body’s capabilities that is psychologically distressing, with some participants distrusting and blaming their body for this reason. Notably, body image distress related to diminished functional impacts is not limited to the endometriosis context, as women with uterine fibroids [35] and polycystic ovarian syndrome [36] report similar distress in relation to their body’s limitations. Theoretically, the limitations endometriosis places on body functionality are a barrier to functionality appreciation, negatively impacting body image [10]. Although it is common for gynecological conditions to negatively impact symbolic gendered identity (i.e., infertility, femininity), what appears different in endometriosis is the chronic, cyclical, and painful symptom profile [37].

Reflecting the curability/controllability dimension of illness representations as described in the Common-Sense Model of Self-Regulation [38], participants reported being constantly challenged by their pain to maintain a sense of control over what is largely an incurable condition. Moreover, avoidance of activity to prevent increasing pain (e.g., no longer exercising) was common. Since behavioral deactivation is considered a precipitant and maintaining factor of clinical depression [39], symptom-related activity avoidance may partially account for the high prevalence of depressed mood in this population [6, 7]. Added to this is the perceived need to hide symptoms and consequent self-silencing that may further contribute to poor mental health outcomes in these individuals [40]. Future research should explore psychological interventions for pain management that have been beneficial in other chronic pain contexts to address body image distress [41, 42], particularly as biomedical intervention alone was cited by the participants as being insufficient in reducing symptoms or re-establishing desired functioning.

The *Needing to Hide Myself* theme was characterized by the desire to socially withdraw due to endometriosis symptoms that made participants feel shameful. This affective response triggered urges to camouflage themselves due to perceiving themselves as being unattractive and less confident physically, particularly due to the symptom of an “endo belly.” Disease-related appearance changes are often considered socially undesirable and thus play an integral role in creating body image–related distress [43]. Participants’ shame around disease-related physical changes can be understood via objectification theory [44]. This approach proposes that female-identified individuals are socialized to learn their value as being based on physical appearance. This in turn results in self-objectification whereby they view their own body from a third-person observer perspective as opposed to attuning to their internal qualities (e.g., kindness) or body functionality [44]. Consistent with prior endometriosis research [8], our findings suggest individuals engage in concealment practices in anticipation that visible abdominal bloating could result in negative social evaluation or ridicule, particularly if an enlarged abdomen was mistaken for pregnancy, reflecting this sense of self-objectification. Participants described hypervigilance in public as they experienced the affective response of fear in relation to their abdominal bloating, which motivated social withdrawal, similar to concealment of excessive facial hair commonly experienced by individuals with polycystic ovarian syndrome [36]. Furthermore, affective body image was salient in the emotions of shame and fear imbued in anticipation of negative social evaluation in the context of sexual intimacy where nudity occurred. Participants reported that in these situations where they could no longer conceal their body via clothing, they felt more vulnerable and unattractive in this context due to visible surgery scarring, shape changes (e.g., weight gain or bloating), and pain; these findings are consistent with prior endometriosis research [8]. Such body surveillance typically occurs when body perceptions do not abide to social norms and are negatively associated with body esteem [45]. Since only a proportion of participants described hypervigilance and concealment practices, it is possible that these individuals may have a high level of appearance investment (i.e., emphasize appearance as part of their self-identity), which in breast cancer has been a risk factor for body image distress [46]. Future clinical interventions for endometriosis affected individuals that focus on mitigating this self-objectification may be helpful in addressing these concerns. In particular, a modified intervention approach similar to that applied to eating disorder treatment may be very suitable for the endometriosis population whereby clinicians enable clients to de-emphasize cognitions and reduce behavioral patterns that reinforce the importance of low weight as a signifier of social acceptability [47]. Likewise, in the endometriosis context, clinicians may be able to target body image distress related to “endo belly” via psychoeducation and cognitive behavioral techniques that will enable the affected individual to reconceptualize their over-emphasis of an idealized feminine shape as antithetical to abdominal bloating and culturally discriminatory beauty standards (i.e., the “perfectly flat stomach” [17]). Furthermore, clinical intervention that specifically targets increasing body functionality appreciation (e.g., [48]) may be beneficial for addressing these concerns in the endometriosis population [20].

Our study provides further evidence that functional body image changes related to infertility negatively impact on a sense of womanhood [8, 49]. Moreover, for those with partners, reassurances did not serve to alleviate this infertility-related body dissatisfaction. This inability to feel reassured by positive partner commentary is similar to findings from qualitative research on lymphedema, a chronic condition with extensive visible bodily impacts, and speaks to the extent to which these negative perceptions are embedded within the individual’s body perceptions [25]. Negative experiences of affective body image were reported when participants were unable to meet their own sexual expectations, and they described guilt and distress for avoiding sexual intercourse to hide their symptoms and prevent intercourse-related pain. Here, the sensory aspects of body image disrupted desire for intimate connection [11]. For health professionals working within the endometriosis context, when body image and sexual concerns are evident, it may be important to seek specialized psychological sexual counselling when a client is distressed [50].

These results corroborate previous findings that endometriosis-related body image concerns differ across age groups [51]. Older participants were more likely to report difficulties related to sexual functioning, weight gain, and fertility, whereas younger participants displayed greater body dissatisfaction characterized by self-directed pejorative language and described more frequent upward comparison to healthy or active women, often feeling misunderstood by their peers. As understood within the framework of the developmental theory of embodiment [52, 53], endometriosis disrupts an individual’s full experiencing of the world, in that attunement to the demands of the disease (e.g., the need to rest) disrupts embodiment, or how they desire their body to be in the world (e.g., fertile, active). These findings are similar to those reported in individuals diagnosed with polycystic ovarian syndrome and uterine fibroids who report feeling envious and distressed about their appearance changes and infertility when they compared themselves to healthy female friends or sisters [35, 36]. These age-related differences highlight the progressive trajectory of endometriosis and the need for research to explore body image concerns across the lifespan, especially as younger women are more likely to have their menstrual-related concerns normalized or dismissed by medical professionals [54].

The *Body as Healer and Teacher* theme exemplifies appreciation of body functionality in the context of having a chronic disease. Within this theme, diagnosis served as an important reparative event in participants’ ability to trust their bodies, concurring with previous research reporting the normalization of menstrual concerns by medical professionals that serves to prolong severe unexplained symptoms and treatment [55]. Diagnosis was seen by our participants as being pivotal in their illness trajectory by validating instincts that their menstrual symptoms are atypical, and providing a sense of relief [31]. Overall, our data suggest diagnosis improved participants’ relationship with their bodies by providing context for their problematic sensory changes (e.g., pain, gastrointestinal issues), thereby helping to mitigate the adverse affective response of anxiety associated with uncertainty about their symptoms.

Additionally, *Body as Healer and Teacher* captured the strategies used to overcome endometriosis-related disturbances to body image, reflecting the “restoring continuity” theme identified by Facchin et al. [8], in that less distressed participants embraced positive lifestyle changes to minimize and manage their problematic symptoms, with some expressing gratitude for the cyclical nature of the disease providing respite. These findings support the theory that improved functionality appreciation [10] corresponds to positive body image, corroborating previous endometriosis research that self-management strategies, such as diet, create a sense of psychological autonomy [56]. These pragmatic attitudes towards endometriosis and pro-active striving to improve their health through a trial-and-error process may have helped participants regain affective control of their body’s sensory reactions. In some accounts, these improvements were juxtaposed with the ineffectiveness of surgical or medical (e.g., hormonal) intervention. Notably for our participants, intentional strategies did not always result in body approval, nor reduce negative body appraisals, but reduced psychological distress by managing uncertainty. Additionally, efforts to control symptoms may reflect the “etiquette of menstruation” whereby all female reproductive experiences culturally demand concealment [24]. As an incurable disease with largely lifestyle-based treatment recommendations, participants’ efforts to self-manage may have taken on particular importance to reduce affective and perceptual body image distress and increase functionality appreciation.

### Limitations and Future Directions

Potential limitations of this study should be considered in light of these results. These data were captured via written survey responses to adhere to COVID-19 social distancing requirements, rather than an in-person interview format, potentially reducing the richness of these data. However, prior work indicates that such online surveys may increase candidness about disclosing intimate details [57], and that for endometriosis populations in particular, disclosure of sensitive information via an online survey is preferred to in-person interviewing [58]. Future research could address this potential limitation by additionally exploring body image in this context using either in-person or Zoom-enabled interviewing approaches. Additionally, it is recommended that future research employs interviewers trained in counselling techniques to conduct interviews to increase the emotional attunement to participant accounts. The online nature of this study and the need for English language competency may have limited participation by those from diverse cultures or with limited socio-economic means, including individuals identifying as being Indigenous. Moreover, despite our recruitment approach aiming to be inclusive, our sample predominantly reflected the experiences of female-identified individuals with only one participant identifying as non-binary. Future research should address these limitations through targeted sampling (e.g., with community centers and clinics that frequently support endometriosis clients) ensuring representation from these diverse communities.

#### Implications

In the context of body image theory [10], it is evident that endometriosis fundamentally disrupts body image in terms of body functionality appreciation, shifting negatively the way their body feels, looks, and behaves. Symptoms of pain and abdominal bloating are part of the definitive nature of the symptomatic disease [59], and widely reported as a concern diminishing functionality appreciation. Nevertheless, future research is needed to investigate appropriate psychosocial interventions that aim to enhance perceptions of body functionality appreciation by challenging aspects that may increase distress, such as self-objectification (i.e., participants’ concern about how others would perceive them; [44]). Quantitative measurement of functionality appreciation, through psychometric measures such as the Functionality Appreciation Scale [16], may be helpful to quantify participant differences in the endometriosis population. These findings further highlight the need for psychological care to be incorporated into routine clinical care for endometriosis. Frequent assessment of aspects of body image such as functionality appreciation could potentially become an aspect of regular care, screening for those with body image concerns who could benefit from body image–focused psychological therapy [17].

This exploratory qualitative analysis evidenced that body image issues are prevalent for individuals with endometriosis affecting their self-image and emotional well-being and involving a breadth of coping strategies. This study offers insight into the body image experience of individuals with endometriosis, specifically their appearance concerns in intimate and private spaces, the barriers they perceived, and how they cope with these physical, psychological, and social challenges. These findings can help inform the design and development of specific strategies to target body image distress in those with endometriosis. Furthermore, these findings highlight how individuals’ perceptions of their body when affected by endometriosis are negatively impacted by the ubiquitous body shape changes associated with this condition. Moreover, the perceived stigma associated with having a “non-normative” body in terms of appearance and functionality is reminiscent of deeply entrenched societal attitudes of ableism that are reflected in these individuals’ shame and embarrassment towards their bodies. Future research is needed to develop appropriate psychoeducational interventions designed to counter these negative attitudes and stereotypes of the “normal” body and to help individuals develop effective strategies to normalize their condition. Moreover, focus on community and societal attitudes is needed to facilitate greater community acceptance of those living with the ever-changing and challenging symptoms of endometriosis that have such extensive adverse impacts on body image.
