# Publisher Correction: “My Body…Tends To Betray Me Sometimes”: A Qualitative Analysis of Affective and Perceptual Body Image in Individuals Living with Endometriosis

**DOI:** 10.1007/s12529-022-10122-5

**Published:** 2022-09-15

**Authors:** Katherine Sayer-Jones, Kerry A. Sherman

**Affiliations:** 1 Centre for Emotional Health, School of Psychological Sciences, Macquarie University, Sydney, Australia; 2 Centre for Emotional Health, School of Psychological Sciences, Macquarie University, Sydney, NSW 2109 Australia

## Publisher Correction: International Journal of Behavioral Medicine https://doi.org/10.1007/s12529-022-10118-1

This article was updated to correct an error in the text made during the production process.

